# 926. Risk factors for post-letermovir cytomegalovirus (CMV) reactivation in allogeneic hematopoietic stem cell transplant (HSCT) recipients

**DOI:** 10.1093/ofid/ofad500.971

**Published:** 2023-11-27

**Authors:** Maria Alejandra Mendoza, Matthew J Thoendel, Raymund R Razonable, Hassan Alkhateeb

**Affiliations:** Mayo Clinic, Rochester, Minnesota; Mayo Clinic, Rochester, Minnesota; Mayo Clinic, Rochester, Minnesota; Mayo Clinic, Rochester, Minnesota

## Abstract

**Background:**

Letermovir (LTMV) is approved for CMV prophylaxis during the first 100 days after HSCT. Thereafter, CMV reactivation (CMVr) may still occur. This study aims to evaluate the incidence and risk factors for any CMVr and clinically significant CMV infection (csCMVi) in patients who received LTMV prophylaxis.

**Methods:**

Retrospective multicenter cohort study of HSCT patients who received 100 days of LTMV prophylaxis from 08/2018 to 09/2022. Categorical and continuous variables were compared with Fisher’s exact and Kruskal-Wallis test respectively. Logistic regression was used to identify risk factors.

**Results:**

Of 115 HSCT patients, 28 had CMVr after LTMV prophylaxis (Table 1), including 21 with csCMVi (Table 2). Of note, 29 (25.2%) patients had breakthrough CMV during LTMV prophylaxis. Among 28 cases of post-LTMV CMVr, DNAemia occurred with a median of 38.5 (31.3 – 47) days after stopping LTMV. The median absolute lymphocyte count (ALC) at diagnosis was 1.29 (0.41 – 2.04) x10^9^cells/L. Median peak CMV DNAemia at was 482 (100.5 – 2600) IU/mL, with a duration of 29 (20- 35) days; none had end organ disease. All were receiving immunosuppression for graft-versus-host disease treatment (n=18) or prophylaxis (n=10).

Risk factors for CMVr and csCMVi are listed in Table 3. For CMVr, univariate analysis showed that CMV D-/R+ status is associated with increased risk, while having CMV D+ donors or acute myeloid leukemia (AML) had lower risk. Multivariable analysis showed D-/R+ status and breakthrough viremia while on LTMV were associated with increased risk.

csCMVi in 21 patients were treated with valganciclovir (n=15) or IV ganciclovir (n=6) for a median duration of 37 (25.5 – 50) days. Univariate analysis showed that acute lymphoblastic leukemia (ALL) and reduced intensity conditioning were associated with increased risk, while AML had lower risk of csCMVi. On multivariable analysis, ALL, and D-/R+ had higher risk while AML had lower risk.

**Figure:**
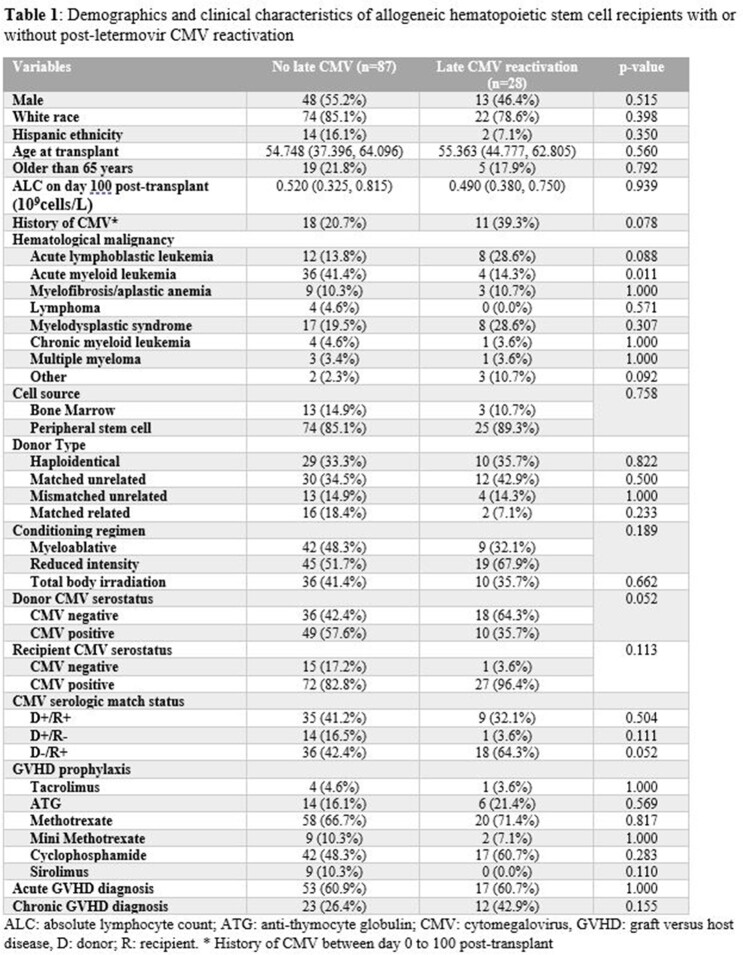

**Figure:**
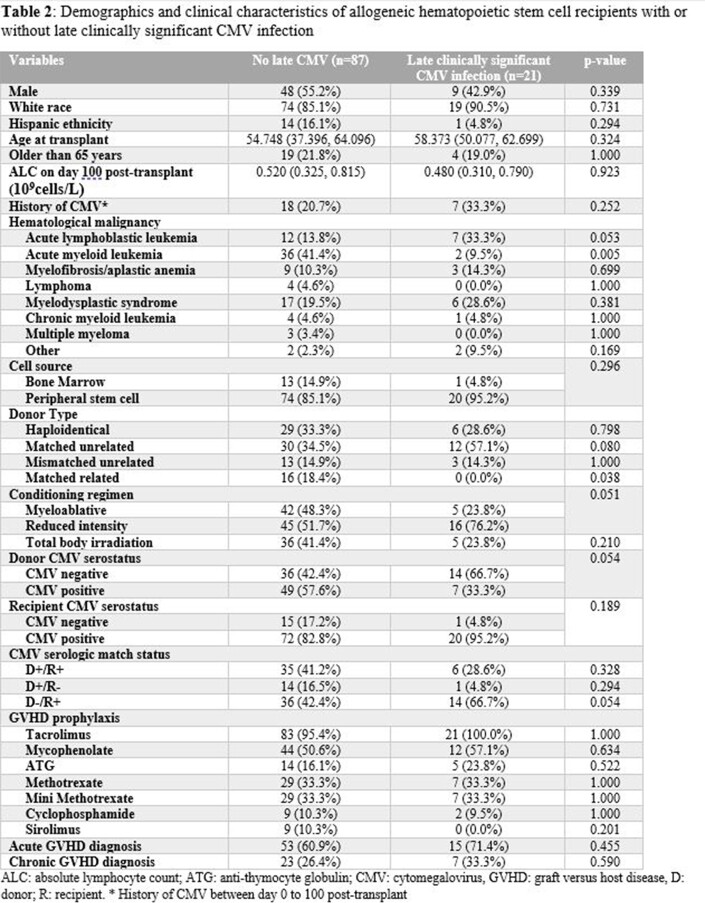

Demographics and clinical characteristics of allogeneic hematopoietic stem cell recipients with or without late clinically significant CMV infection

**Figure:**
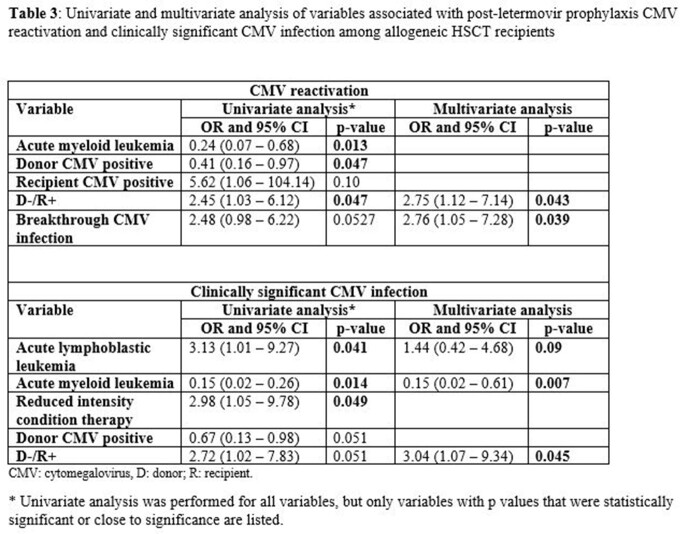

**Conclusion:**

One in four allogeneic HSCT patients who received LTMV developed post-prophylaxis CMVr within 100 to 200 days post-transplant. CMV D-/R+ status had a higher risk for CMVr and csCMVi after LTMV discontinuation. Patients with AML had a lower risk for CMVr and csCMVi.

**Disclosures:**

**Raymund R. Razonable, MD**, Allovir: Endpoint Adjudication Committee|American Society of Transplantation: Board Member|Gilead: Grant/Research Support|Novartis: DSMB|Regeneron: Grant/Research Support|Roche: Grant/Research Support

